# Functions of Some Capsular Polysaccharide Biosynthetic Genes in *Klebsiella pneumoniae* NTUH K-2044

**DOI:** 10.1371/journal.pone.0021664

**Published:** 2011-07-12

**Authors:** Jin-Yuan Ho, Tzu-Lung Lin, Chun-Yen Li, Arwen Lee, An-Ning Cheng, Ming-Chuan Chen, Shih-Hsiung Wu, Jin-Town Wang, Tsung-Lin Li, Ming-Daw Tsai

**Affiliations:** 1 Institute of Biological Chemistry, Academia Sinica, Taipei, Taiwan; 2 Genomics Research Center, Academia Sinica, Taipei, Taiwan; 3 Chemical Biology and Molecular Biophysics Program, Taiwan International Graduate Program, Institute of Biochemistry, Academia Sinica, Taipei, Taiwan; 4 Institute of Biochemical Science, National Taiwan University, Taipei, Taiwan; 5 Department of Microbiology, National Taiwan University College of Medicine, Taipei, Taiwan; 6 Institute of Bioinformatics and Structure Biology, National Tsing Hua University, Hsinchu, Taiwan; 7 Department of Internal Medicine, National Taiwan University Hospital, Taipei, Taiwan; Monash University, Australia

## Abstract

The growing number of *Klebsiella pneumoniae* infections, commonly acquired in hospitals, has drawn great concern. It has been shown that the K1 and K2 capsular serotypes are the most detrimental strains, particularly to those with diabetes. The K1 *cps* (capsular polysaccharide) locus in the NTUH-2044 strain of the pyogenic liver abscess (PLA) *K. pneumoniae* has been identified recently, but little is known about the functions of the genes therein. Here we report characterization of a group of *cps* genes and their roles in the pathogenesis of K1 *K. pneumoniae*. By sequential gene deletion, the *cps* gene cluster was first re-delimited between genes *galF* and *ugd*, which serve as up- and down-stream ends, respectively. Eight gene products were characterized *in vitro* and *in vivo* to be involved in the syntheses of UDP-glucose, UDP-glucuronic acid and GDP-fucose building units. Twelve genes were identified as virulence factors based on the observation that their deletion mutants became avirulent or lost K1 antigenicity. Furthermore, deletion of *kp3706*, *kp3709* or *kp3712* (Δ*wcaI*, Δ*wcaG* or Δ*atf*, respectively), which are all involved in fucose biosynthesis, led to a broad range of transcriptional suppression for 52 upstream genes. The genes suppressed include those coding for unknown regulatory membrane proteins and six multidrug efflux system proteins, as well as proteins required for the K1 CPS biosynthesis. In support of the suppression of multidrug efflux genes, we showed that these three mutants became more sensitive to antibiotics. Taken together, the results suggest that *kp3706*, *kp3709* or *kp3712* genes are strongly related to the pathogenesis of *K. pneumoniae* K1.

## Introduction

Bacterial pathogenicity has been shown to be due to different causes, including the structures of capsular polysaccharides (CPS; the K antigen), lipopolysaccharide (LPS; the O antigen), secreted toxins, drug resistance, and genetics [1], [2], [3], [4], [5]. *Klebsiella pneumoniae* is an opportunistic pathogen of the *Enterobacteriaceae* and usually causes pneumonia or urinary tract infections [6]. In addition, the hospital outbreak of multidrug resistant *Klebsiella spp.*, especially the so-called extended-spectrum beta lactamase (ESBL) and *Klebsiella pneumoniae* carbapenemase (KPC) subtypes, has draw much attention in recent years [6], [7], [8].

The CPS of *K. pneumoniae* is complex acidic polysaccharide consisting of repeating units of 3–6 sugars. The type of sugars seems to correlate with the virulence, and 78 capsule types have been identified [9]. In the past two decades, a number of *K. pneumoniae* strains have been found to cause primary pyogenic liver abscess (PLA) [10], [11], [12], [13], with the capsular serotype K1 being the most virulent [14], [15]. The K1 structure has been reported previously (lacking the acetyl-decoration on fucose) to possess two unique features - a fucose subunit (also found only in K54 and K63), and a unique cyclic 2,3-(*S*)-pyruvate appendix differing from a commonly seen 4,6-(*R*)-pyruvate in CPS repeat units [16], [17].

**Figure 1:**
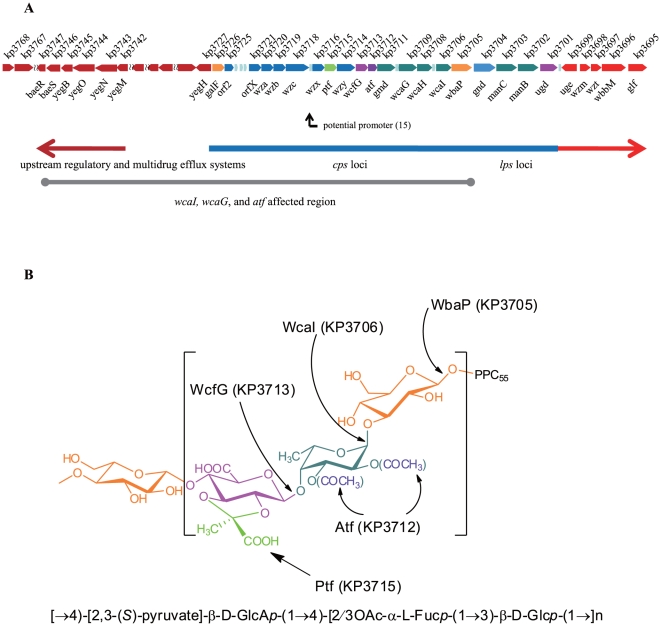
Structure of NTUH-K2044 CPS trisaccharide and summary of our work. **A**, The upper scheme shows the annotated *cps* cluster and its upstream and downstream genes reported previously. In the middle scheme, the blue bar indicates the CPS gene cluster re-delimited in this work, from *gnd* (*kp3701*) to *galF* (*kp3726*). In the lower scheme, the grey bar indicates the suppressed gene region [from *wbaP* (*kp3705*) to *fbaB* (*kp3767*)] in Δ*wcaI*, Δ*wcaH*, and Δ*atf* deletion strains identified in this work. (The color codes used in the *cps* locus represent the following genes: orange - biosynthesis of glucose in the trisaccharide formation; dark green - biosynthesis of fucose; purple - decoration of acetyl moiety; pink - biosynthesis of glucuronic acid; light green - decoration of pyruvyl moiety; dark blue – regulation of the *cps* locus and assembly of the long train CPS.) **B**, Re-determined CPS trisaccharide structure.

It has been reported previously that *magA* (mucoviscosity associated gene A) in the *cps* locus of NTUH-K2044, a PLA-causing serotype K1 strain from National Taiwan University Hospital [18], is associated with the hypermucoviscosity of the strain and considered a virulence factor [18]. A 31-Kb fragment that covers regions from genes *galF* (*kp3726*) to *ugd* (*kp3701*) was further identified genetically as the K1 *cps* cluster (**Figure 1A**) [15]. Recently, the whole genome for this PLA strain has been sequenced and annotated, where a previously unidentified acetyl transferase gene was shown in the *cps* cluster [19]. Our collaborators have re-determined the CPS structure (**Figure 1B**), and demonstrated that the acetylation occurs on either C2 or C3 of the fucose (unpublished results). In this work, we took a systematic approach to study the biosynthetic genes of the CPS of NTUH-K2044. Our goal is to identify the functions of the genes and their relevance to pathogenicity.

## Results and Discussion

### Identification of specific genes as virulence factors

**Figure 2:**
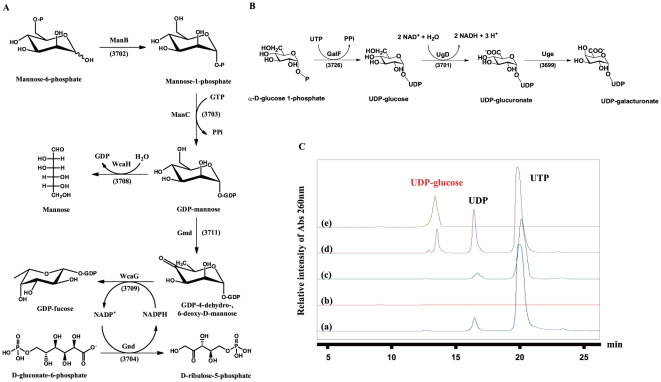
Characterization of the biosynthetic pathway of trisaccharide building blocks. **A**, Biosynthetic pathway of GDP-fucose. **B**, Biosynthetic pathway of UDP-glucose and UDP- galacturonic acid. **C**, HPLC traces of GalF reaction, (a) starting substrates, 1 mM glucose-1-phosphate and 1 mM UTP; (b) 1 mM glucose-1-phosphate, in the presence of GalF; (c) 1 mM UTP, in the presence of GalF; (d) 1 mM glucose-1-phosphate and 1 mM UTP, in the presence of GalF; (e) 1 mM commercial UDP-glucose as a product standard. The enzyme reactions were carried out in a buffer solution (50 mM Tris-HCl, pH 8.0, 5 mM Mg_2_SO_4_) at 37°C for 4 hr.

We examined the functions of individual genes in CPS biosynthesis, aiming to verify the *cps* locus annotation (**Figure 1A**) and to establish the connection between the chemical structure and the biosynthetic genes in their contributions to pathogenicity. First, the genes for NDP-sugar biosyntheses (*manB*, *manC*, *wcaH*, *gnd*, *wcaG*, *gmd*, *galF*, *ugd* and *uge*) (**Figure 2A–B**) were functionally verified by over expressing and purifying recombinant enzymes and identifying their products by high-pressure liquid chromatography (HPLC) and mass spectrometry. The HPLC analyses of the reaction catalyzed by GalF are shown in **Figure 2C** as an example. The rest of the HPLC analyses are shown in **Figures S1 and S2**. The kinetic parameters of Ugd and Gnd are shown in **Table S1**, and the specificity of ManC and GalF are shown in **Tables S2, S3**. Detailed procedures are described in **Results S1**.

**Figure 3:**
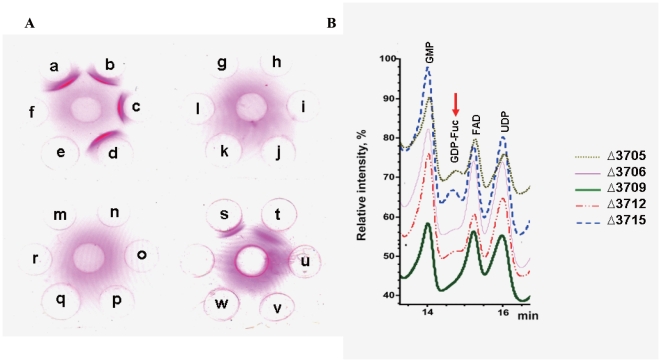
Characterization of the in-frame deletion mutants (dxxxx), complementation strains of the deletion mutants (cxxxx), and insertion mutants (ixxxx). **A**, Double immunodiffusion assay for the K1 serotype. a: wild-type, b: d3695, c: d3699, d: d3704, e: d3713, f: d3714, g: i3705, h: d3705, i: i3706, j: d3706, k: i3709, l: d3709, m: d3712, n: i3715, o: d3715, o: d3718, q: d3719, r: d3720, s: wild-type, t: d3708, u: c3706, v: c3709, w: c3712. Only wild-type, d3695, d3699, d3704, and d3708 remained K1 positive. **B**, HPLC analysis of NDP-sugars. All peaks were determined by the standard retention time and confirmed by MS (**Figure S3**). The GDP-fucose peak is indicated by the red arrow. The data for the wild-type was not obtained due to the high mucoviscosity, which prohibited extraction of metabolites.

Then in-frame deletion mutants were constructed using a modified pKO3 system [20] for *ugd*, *gnd*, *wbaP*, *wcaI*, *wcaH*, *wcaG*, *gmd*, *atf*, *wzy (magA)*, *ptf*, *wzc*, *wzb*, and *wza* in the proposed *cps* locus (**Tables 1**). All mutants were obtained except Δ*ugd* and Δ*gmd*. Based on both anti K1 serum test by double immunodiffusion assay (**Figure 3A**) and string test [15], all mutants obtained (except Δ*gnd* and Δ*wcaH*) lost the K1 serotype and mucoviscosity while remaining O1 serotype positive (**Table 2**), suggesting that these mutants produce little or no CPS. Moreover, deletion of *glf* and *uge* in the proposed *lps* locus led to loss of O1 serotype, confirming the proposed boundary between *cps* and *lps* loci shown in **Figure 1A**.

**Table 1:** Bacterial strains and plasmids used in this study.

Bacterial strains or plasmids	Genotype or prevalent description	References or sources
***K. pneumoniae*** ** strains**		
NTUH-K2044	Clinical isolate; the parent strain for generate isogenic mutants	(18)
i3705	km cassette in *wbaP* ORF	(15)
i3706	km cassette in *wcaI* ORF	(15)
i3709	km cassette in *wcaG* ORF	(15)
i3714	km cassette in *wzy* ORF	(15)
i3715	km cassette in *ptf* ORF	(15)
d3695	*glf* deletion strain in NTUH K2044 background	present study
d3699	*uge* deletion strain in NTUH K2044 background	present study
d3704	*gnd* deletion strain in NTUH K2044 background	present study
d3705	*wbaP* deletion strain in NTUH K2044 background	present study
d3706	*wcaI* deletion strain in NTUH K2044 background	present study
d3708	*wcaH* deletion strain in NTUH K2044 background	present study
d3709	*wcaG* deletion strain in NTUH K2044 background	present study
d3712	*atf* deletion strain in NTUH K2044 background	present study
d3713	*wcfG* deletion strain in NTUH K2044 background	present study
d3714	*wzy* deletion strain in NTUH K2044 background	present study
d3715	*ptf* deletion strain in NTUH K2044 background	present study
d3718	*wzc* deletion strain in NTUH K2044 background	present study
d3719	*wzb* deletion strain in NTUH K2044 background	present study
d3720	*wza* deletion strain in NTUH K2044 background	present study
d3742	*yegM* deletion strain in NTUH K2044 background	present study
c3706	complement of *wcaI* in d3706	present study
c3709	complement of *wcaG* in d3709	present study
c3712	complement of *atf* in d3712	present study
***E. coli*** ** strains**		
DH5α		
BL21 star		
BL21 codon plus		
**Plasmids**		
pET28a	expression cloning	Novagen
pGEM-T easy-km	pGEM-T easy was inserted with Km cassette from pUC4K into NdeI site for *trans* complementation	Present study
pKO3-km	pKO3-derived plasmid, with an insertion of Km resistance cassette from pUC4K into AccI site	(36)
pCRIITOPO-CAT	for cloning the complement plasmid	(15)

**Table 2:** K1 antigenicity, O antigenicity, and LD50 values of all strains in this study.

*K. pneu.* Strains	K1 Antigenicity	O Antigenicity	LD_50_ (CFU) [N]^a^
NTUH-K2044	+	+	<10^2^ [4]
i3705	−	ND	ND
i3706	−	ND	ND
i3709	−	ND	ND
i3714	−	ND	ND
i3715	−	ND	ND
d3695	+	−	ND
d3699	+	−	ND
d3704	+	+	ND
d3705	−	+	ND
d3706	−	+	ND
d3708	+	+	ND
d3709	−	+	ND
d3712	−	+	>10^7^ [4]
d3713	−	+	ND
d3714^b^	−	+	>10^7^ [4]
d3715	−	+	>10^7^ [4]
d3718	−	+	ND
d3719	−	+	ND
d3720	−	+	ND
c3706	−	ND	ND
c3709	−	ND	ND
c3712	−	ND	ND

ND: not determined,

a mice numbers for inoculation,

b (Ref. 15).

Since the deletion mutants make little or no CPS, they are expected to lose pathogenicity also. Animal inoculation experiments were performed for three mutants as examples. As shown in **Table 2**, the results indicate that deletion of the acetyltransferase gene (Δ*atf* or Δ*3712*), the pyruvyltransferase gene (Δ*ptf* or Δ*3715*), or the mucoviscosity associated gene (Δ*magA*, Δ*wzy*, or Δ*3714*) was sufficient to cause a significant decrease in the virulence of the NTUH-K2044 strain (LD_50_>10^7^ CFU in intra-peritoneal infection, relative to <10^2^ for WT).

These results taken together have verified the functions of individual genes and proteins. Even though the CPS itself can be considered as a virulence factor, our results suggested that the individual genes or proteins responsible for CPS synthesis can also be considered as virulence factors, and thus are potential targets for designing inhibitors against the pathogen. We further examined the properties of the deletion mutants as described below.

### Discovery of a broad gene silencing effect related to fucose biosynthesis

We first examined the deletion mutants at the transcriptional level by Q-PCR. Surprisingly, expressions for genes between *wcaI* and *galF*, most of the genes in the *cps* gene cluster, were found completely silenced in d3706, d3709 and d3712 (**Table 3**), while they expressed normally (except the deleted gene) in the other deletion mutants (**Table S4**). To estimate the scopes of the gene silencing effect, genes upstream of the *cps* locus were also examined and found to be silenced up to *kp3767* in these three mutants (**Table 4** **, left 4 lanes**). The total region influenced by the effect was about 70 Kb, including 15 of the 20 *cps* genes and 37 upstream genes (**Figure 1A** **and Table S5**). Interestingly, the functions of these three genes are all related to the fucose residue of the trisaccharide repeat unit - WcaI (KP3706) is likely the fucosyl transferase, WcaG (KP3709) is responsible for GDP-fucose synthesis, and Atf (KP3712) is for fucose acetylation.

**Table 3:** Gene expression of mutant strains.

Strains	d3706	d3709	d3712	d3713	d3715
**Genes**					
*kp3689*	0.13	0.54	0.99	0.53	0.27
*kp3693*	0.11	0.47	0.73	0.78	0.28
*kp3694*	0.12	0.38	0.69	0.52	0.27
*kp3695*	0.23	0.99	0.88	0.82	0.44
*kp3696*	0.15	0.64	0.59	0.98	0.34
*kp3699*	0.22	1.02	0.76	1.14	0.48
*kp3701*	0.10	0.32	0.10	0.86	0.54
*kp3702*	0.10	0.44	0.18	1.06	0.62
*kp3703*	0.11	0.35	0.09	0.60	0.61
*kp3704*	0.17	0.56	0.50	0.70	0.61
*kp3705*	**0.00**	**0.00**	**0.00**	0.62	0.53
*kp3706*	**0.00**	**0.00**	**0.00**	1.12	0.50
*kp3708*	**0.00**	**0.00**	**0.00**	0.80	0.56
*kp3709*	**0.00**	**0.00**	**0.00**	0.65	0.78
*kp3711*	**0.00**	**0.00**	**0.00**	0.40	0.75
*kp3712*	**0.00**	**0.00**	**0.00**	0.36	0.63
*kp3713*	**0.00**	**0.00**	**0.00**	**0.00**	0.66
*kp3714*	**0.00**	**0.00**	**0.00**	0.45	0.59
*kp3715*	**0.00**	**0.00**	**0.00**	0.52	**0.00**
*kp3716*	**0.00**	**0.00**	**0.00**	0.86	0.81
*kp3718*	**0.00**	**0.00**	**0.00**	0.37	0.81
*kp3719*	**0.00**	**0.00**	**0.00**	0.56	0.89

Genes that are no expression are highlighted in bold black.

**Table 4:** Gene expression of deletion mutants and complementation strains.

Strains	d3706	d3708	d3709	d3712	c3706	c3709	c3712
Genes							
*kp3699*	0.22	1.04	0.99	0.59	0.27	0.46	0.32
*kp3701*	0.10	0.43	0.34	0.25	0.24	0.43	0.16
*kp3702*	0.10	0.32	0.40	0.18	0.55	0.29	0.11
*kp3703*	0.11	0.26	0.41	0.18	0.07	0.05	0.12
*kp3704*	0.17	0.74	0.59	0.30	0.23	1.10	0.23
*kp3705*	**0.00**	0.26	**0.01**	**0.00**	**0.01**	**0.00**	**0.07**
*kp3706*	**0.00**	0.17	**0.00**	**0.00**	**0.93** a	**0.00**	**0.00**
*kp3708*	**0.00**	**0.00**	**0.00**	**0.00**	**0.00**	**0.00**	**0.00**
*kp3709*	**0.00**	0.23	**0.00**	**0.00**	**0.00**	**2.58** a	**0.01**
*kp3711*	**0.00**	0.26	**0.00**	**0.00**	**0.00**	**0.00**	**0.00**
*kp3712*	**0.00**	0.27	**0.00**	**0.00**	**0.00**	**0.00**	**4.92** a
*kp3713*	**0.00**	0.26	**0.00**	**0.00**	**0.00**	**0.00**	**0.00**
*kp3714*	**0.00**	0.18	**0.01**	**0.01**	**0.00**	**0.03**	**0.01**
*kp3715*	**0.00**	0.28	**0.00**	**0.01**	**0.00**	**0.00**	**0.01**
*kp3716*	**0.00**	0.20	**0.00**	**0.00**	**0.00**	**0.00**	**0.01**
*kp3718*	**0.00**	0.31	**0.00**	**0.00**	**0.00**	**0.00**	**0.01**
*kp3719*	**0.00**	0.32	**0.00**	**0.01**	**0.00**	**0.00**	**0.01**
*kp3720*	**0.00**	0.24	**0.00**	**0.00**	**0.00**	**0.00**	**0.00**
*kp3721*	**0.00**	0.33	**0.00**	**0.00**	**0.00**	**0.00**	**0.00**
*kp3725*	**0.00**	0.19	**0.00**	**0.00**	**0.00**	**0.00**	**0.00**
*kp3726*	**0.01**	0.22	**0.01**	**0.00**	**0.01**	**0.01**	**0.01**
*kp3731*	**0.00**	0.42	**0.00**	**0.00**	**0.00**	**0.00**	**0.00**
*kp3736*	**0.01**	0.68	**0.01**	**0.01**	**0.00**	**0.00**	**0.00**
*kp3744*	**0.00**	0.57	**0.00**	**0.00**	**0.00**	**0.00**	**0.00**
*kp3751*	**0.03**	0.69	**0.01**	**0.01**	**0.00**	**0.00**	**0.01**
*kp3759*	**0.03**	0.43	**0.00**	**0.00**	**0.00**	**0.00**	**0.00**
*kp3767*	**0.00**	1.26	**0.00**	**0.00**	**0.00**	**0.00**	**0.00**
*kp3768*	2.31	0.44	0.48	1.17	2.67	0.62	0.00
*kp3770*	0.03	0.18	0.13	0.08	0.10	0.35	0.73

Figures highlighted in bold black represent the genes subject to the gene silencing effect.

a Figures represent restoration of gene expression by a given complement plasmid.

### Further support for the broad gene silencing effect

That the broad gene silencing effect is novel and real is further supported by three experimental approaches: (a) Complementation experiments with plasmids carrying the deleted gene restored only the expression of the deleted gene, not the other silenced genes (**Table 4** **, right 3 lanes**). This result suggests that the silencing effect is caused by changes at the level of genomic DNA, not simply due to protein expression. (b) The effect of gene silencing was observed from fucose-related metabolites. Analyses of cell fluids extract showed that GDP-Fuc were hardly detectable in these three mutants, while clearly present in two control mutants d3705 and d3715 (**Figure 3B**). The results were also verified by MS analyses (**Figure S3**). This result supports the silencing effect since only KP3709 is involved in GDP-fucose biosynthesis; the functions of KP3706 and KP3712 occur after formation of GDP-fucose and their deletion should not have affected the production of GDP-fucose if there were no silencing effect.

We then examined whether the broad silencing effect described above could be due to any known regulatory mechanism. The first to consider was whether the observed silencing effect is a form of well known “polar effect” [21] (suppression of a small number of downstream genes) often observed for insertion mutants. Even though the in-frame deletion mutants in our studies were designed to avoid polar effects [20], some insertion mutants from our previous work [15] were examined to see if they display the broad silencing effect as described above for the deletion mutants. As shown in **Table 5**, polar effects were observed for i3706, i3709, which are distinctly different from the broad silencing effect in the deletion mutants. Furthermore, the K1 serotype of insertion mutants can be restored by complementation as shown previously [15], but not that of the three deletion mutants (**Figure 3A-u∼w** **and** **Table 4**).

**Table 5:** Gene expression of insertion mutant strains.

Strains	i3705	i3706	i3709
Genes			
*kp3705*	**0.325**	**0.061**	**0.129**
*kp3706*	2.313	**0.062**	**0.160**
*kp3708*	2.567	1.064	**0.071**
*kp3709*	2.567	1.007	**0.022**
*kp3711*	2.378	0.683	0.953
*kp3712*	2.479	1.094	1.050
*kp3713*	2.990	0.722	0.889
*kp3714*	3.117	0.908	1.094
*kp3715*	4.170	1.141	1.580
*kp3716*	5.389	1.636	2.144
*kp3718*	2.514	0.559	0.774
*kp3719*	5.657	2.028	1.892

Figures highlighted in bold black are the genes subject to the polar effect.

Several other known regulatory mechanisms are related to the *cps* gene cluster, but not involved in the expression of upstream genes: (i) regulators of capsule synthesis (*rcs*) [22], which sense the extracellular signals then regulate the expression of *cps* gene cluster; (ii) the transcriptional antiterminator *rfa*H [1], [3], which recognizes Just Upstream of Many Polysaccharide Starts (JUMPstar) to ensure the expression of distal genes; (iii) *wzb*/*wzc* [1], which are believed to be involved in the CPS polymerization; and (iv) *rmpA2*, which is a transcriptional activator and its absence would only lower the capsule production [15]. The expression level of these regulatory genes shows no significant difference between wild-type and mutants.

Small RNA (sRNA) is specifically used to represent bacterial non-coding RNA. Since it has been suggested that sRNA [23] effects could be dependent on the temperature of bacterial growth [24] or the state of growth [25], we examined the gene silencing effect at different temperatures (25, 42 and 45°C) (**Table S6**) and at the stationary phase of the growth (**Table S7**). The results indicate that these factors did not affect the broad gene silencing effect observed for the three deletion mutants.

### Synergy between the fucose-related virulence factors and drug resistance

**Figure 4:**
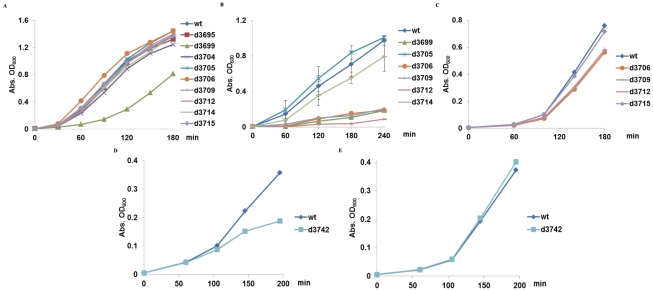
Growth curves of wild-type and mutants and effects of antibiotics. **A**, Wild-type and all deletion mutants obtained in this study, under normal growth conditions. The mutant of LPS gene *kp3699* was used for comparison. **B** and **C**, Effects of zeocin (50 µg/ml) and erythromycin (20 µg/ml) on selective mutants. These two antibiotics selectively slowed down the growth of the three mutants d3706, d3709, and d3712. **D** and **E**, Effects of zeocin and erythromycin on WT and the Δ*yegM* mutant. All data are shown as the mean ± SEM from three experiments.

To test whether the broad gene silencing effect is related to the pathogenicity of the NTUH-K2044 strain, we examined possible functions of the silenced genes. Importantly, the 37 genes upstream of the *cps* locus affected by the gene silencing effect include many regulators and multidrug efflux genes (from *kp3742* to *kp3747*) (**Figure 1A** **and Table S5**). Since multidrug efflux pumps are known to contribute to drug resistance in Gram-negative bacteria [26], this finding led us to predict that the three mutants d3706, d3709, and d3712 would be less drug-resistant to some of the antibiotics. As shown in **Figure 4A**, in the absence of antibiotics there were no significant differences in growth rates between K2044 and its mutants, confirming that CPS is important for pathogenicity but not for growth (Δ*uge* or d3699, a slow-growing mutant, was used as a positive control as Uge is involved in the biosynthesis of LPS). Then we examined the effects of various antibiotics on the growth rates of these strains. Two of the antibiotics tested, zeocin (a member of the bleomycin/phleomycin family of antibiotics known to bind and cleave DNA) (**Figure 4B**) and erythromycin (a macrolide family of antibiotics known to interfere with protein synthesis) (**Figure 4C**) showed significant inhibition against Δ*wcaI*, Δ*wcaG*, and Δ*atf* (d3706, d3709, and d3712, respectively) but had limited effect against wild-type and the other mutants. In contrast, tetracycline, sulfamethoxazole, ciprofloxacin, and geneticin, which are classified as members of tetracyclines, sulfonamides, quinolones, and aminoglycosides antibiotics, respectively, did not show specific effects on the three mutants (**Figure S4A–S4D**).

If the effects of zeocin or erythromycin on the three deletion mutants are indeed caused by silencing of multidrug efflux genes, we should be able to identify a single efflux gene, construct the deletion mutant of the single gene, and show that the mutant is sensitive to the antibiotics. To test this possibility, a *kp3742* (*yegM*) deletion mutant was constructed. The inhibition assays showed that the growth of the Δ*yegM* strain was inhibited by zeocin (Figure 4D) but not erythromycin (Figure 4E) or the other antibiotics (not shown).

There has been growing evidence that fucose [27] and acetylation [28], [29], [30] are important factors in bacterial pathogenicity and in other diseases. For example, *Helicobacter pylori* is a well known primary cause of gastritis, duodenal ulcer, and gastric cancer, which infects about one-half of human population. Liu et al. demonstrated the important connection between human L-fucosidase (FUCA2) and *H. pylori* on the adhesion pathogenesis and escape of host surveillance [31]. In addition, Coyne et al. also demonstrated that bacteroides in mammalian intestine use a mammalian-like pathway to decorate numerous capsular polysaccharides and glycoproteins with fucose [32]. No mechanism has been suggested about the special roles of fucose. Our results add an example in the potential biological significance of fucose, and we have discovered some link between fucose biosynthetic genes and gene silencing. However the specific functions of fucose or fucose biosynthetic genes remain to be further investigated.

In summary, our results on the CPS of the NTUH-K2044 strain of the PLA *K. pneumoniae* add to the emerging evidence that fucose [32] and O-acetylation [28] are important factors in bacterial pathogenicity, and our genetic analyses of deletion mutants uncover a possible link between three fucose-related genes and the multidrug efflux genes via an unprecedented broad gene networking. This phenomenon potentially represents a newly uncovered pathogenesis mechanism. These results led us to hypothesize that the broad silencing effects observed for the three in-frame deletion mutants are caused by DNA structure alteration in the deletion mutants of the three fucose-related genes, and that the silencing effects may be relevant to the specific roles of the fucose and its acetylation in the pathogenicity of the strain. The validity of the hypothesis, and the mechanism of the broad silencing effect, are important subjects for future studies.

## Materials and Methods

### Bacterial strains and plasmid vectors

Bacterial strains and plasmids used in this study are listed in **Table 1**. Clinically isolated *K. pneumoniae* strains were collected at National Taiwan University Hospital (NTUH) [18].

### Serum resistance assay and animal inoculation

The serum resistances of *K. pneumoniae* strains were determined as previously described [33]. Female BALB/cByl 5-week-old mice were used for inoculation. BALB/cByl mice were bred and housed in specific pathogen–free rooms within animal care facilities of the Laboratory Animal Center at the National Taiwan University College of Medicine (NTUCM) with free access to food and water. All procedures were approved by the National Taiwan University College of Medicine and College of Public Health Institutional Animal Care and Use Committee (IACUC), following with the recommendations in the Guide for the Care and Use of Laboratory Animals of the National Institutes of Health and Taiwanese Animal protection act. IACUC approval number: 20060139. *K. pneumoniae* inoculation consisting of 10^2^–10^6^ mid-logarithmic growth phase CFUs were diluted in 100 µl normal saline and injected intraperitoneally [34], [35]. Four mice were used to test the effects of each inoculum. After inoculation, the mice were observed for 30 days. The LD_50_ was calculated using the method established by Reed and Muench [35].

### Construction of *K. pneumoniae* deletion mutant strains

The deletion mutants were generated using a modified pKO3-Km vector that contained a temperature-sensitive origin of replication and markers for positive and negative selection for chromosomal integration and excision [36], [37]. The genes and its flanking regions were amplified by PCR using primers listed in **Table S8** and cloned into a pGEM-T easy vector. The deletion fragment was generated by inverse PCR using primers listed in **Table S8**. The deletion fragments described above were cloned into a *Not*I site of a pKO3-Km plasmid separately [36]. The resulting constructs were then electroporated into wild-type strain. The transformants were cultured at 43°C. Five colonies were picked in 1 ml LB broth followed by serial dilution and plating onto LB plates containing 5% sucrose cultured at 30°C. Colonies sensitive to kanamycin were screened and confirmed by PCR using appropriate combinations of primers, and DNA sequencing.

### Trans-complementation

The *wcaG*, *wcaI*, and *atf* genes were amplified by PCR using primers (**Table S1**) and cloned into a pGEM-T Easy-Km plasmid [9]. These plasmids were transformed into their corresponding isogenic mutant strains by electroporation [38]; for selection of complementation strains, LB agar plates were supplemented with 50 µg/ml kanamycin or 100 µg/ml chloramphenicol.

### String test

The mucoviscosity of *K. pneumoniae* was determined by a string test as previously described [33]. The bacteria were cultured on 5% sheep blood agar plates overnight. Colonies were tested by inoculation loop. If the colony could be stretched to a string longer than 0.5 cm then it was defined as positive string test; otherwise as negative.

### K1 and O serotyping

Capsule and lipopolysaccharide were purified as previously described [15]. The K1 serotyping was performed by the double immunodiffusion assay using a serotype K1–specific antiserum (Statens Serum Institute) as previously described [15]. After separation in 12% SDS-PAGE gel, the O antigen was detected by sera from mice infected with a capsule-deficient *magA* deletion mutant.

### Quantitative PCR

1 µg of extracted RNA was reverse transcribed into cDNA in 20 µl reaction as the manufacturer protocol (Roche, US). 20 µl of cDNAs were then diluted into 1 ml. 5 µl of diluted cDNA samples were added into the Q-PCR reaction plate, and also the 10 µl of reaction solution. The analysis of Q-PCR result is that each gene was first normalized with 23S RNA. Each gene in each strain was then normalized with wild-type to obtain the relative gene expression pattern. The primers used for Q-PCR are listed in **Table S9**.

### Gene cloning, protein purification, and functional assay

Genes (*uge*, *ugd*, *manB*, *manC*, *gnd*, *wcaH*, *wcaG*, *gmd*, and *galF*) were cloned from NTUH K-2044 genomic DNA into pET28a (his-tagged). Proteins (Uge, Ugd, ManB, ManC, Gnd, WcaH, WcaG, Gmd, and GalF) were over-expressed in *E. coli*. BL21 star (DE3). His-tagged protein purification was followed by PROBOND (Invitrogen, US) manufacturer's protocol. Purified proteins were concentrated to 1 mg/ml then 10 µl of protein together with 10 µl of 10 mM substrates (in the figure legend) were added in reaction buffer, 50 mM Tris-HCl (pH 8.0), 5 mM MgCl_2_ in final 100 µl solution. The reaction solution was extracted by chloroform to remove the proteins, then analyzed by HPLC (Angilent, HP1100, US) with ammonium formate gradient.

### Extraction and separation of nucleotides and sugar nucleotides

For isolation of soluble fractions containing sugar nucleotide/nucleotides from *K. pneumoniae* strains, cells were collected when OD_600_ was 1.0. The cells were harvested by centrifugation at 5000 rpm for 30 min at 4°C. Cell pellets were re-suspended in 1 ml phosphate-buffered saline (PBS, pH 7.4), followed by addition of a mixture chloroform/methanol (1∶2) and vortexing for 10–15 min. The mixture was then centrifuged at 4000 rpm for 15 min, the pellet was removed and mixed with chloroform and ddH_2_O (1∶1), and centrifuged again. The upper phase containing soluble components were collected and dried under air. It was redissolved in distilled water and further purified by Amicon filter (YM-10 cut-off), and the filtrate was collected and monitored by anion exchange HPLC using ammonium formate. The peaks were identified by comparing the retention times and MS with known standards.

### Growth curves of WT and mutants and effects of antibiotics

NTUH-K2044 and knock-out strains were grown in LB broth at 37°C. For growth curves, log phase cultures were diluted to OD_600_ = 0.005 in LB broth with desired antibiotics. The growth curve was plotted by measuring OD_600_ periodically.

## Supporting Information

Results S1 **Characterization of enzymes for the synthesis of UDP-glucose (UDP-Glc), UDP-glucuronic acid (UDP-GlcA), UDP-galacturonic acid (UDP-GalA), GDP-mannose (GDP-Man) and GDP-fucose (GDP-Fuc).**
(DOC)

Figure S1 **HPLC traces.** **A,** Ugd (KP3701) reaction buffers containing: (a) UDP-glucose+NAD^+^, (b) UDP-glucose+KP3701, (c) NAD^+^+KP3701 (d) UDP-glucose+NAD^+^+KP3701. **B,** Uge (KP3699) reaction buffers containing: (a) UDP-glucuronate+Mg^2+^, (b) UDP-glucuronate+Mg^2+^+KP3699.
(EPS)

Figure S2 **HPLC traces.** **A,** ManC (KP3703) reaction buffers containing: (a) mannose-1-phosphate+GTP+Mg^2+^, (b) mannose-1-phosphate+Mg^2+^+KP3703, (c) GTP+Mg^2+^+KP3703, (d) mannose-1-phosphate+GTP+Mg^2+^+KP3703, (e) GDP-mannose standard. **B,** ManB (KP3702) and ManC (KP3703) reaction buffers containing: (a) mannose-6- phosphate+GTP+Mg^2+^+KP3702, (b) mannose-1-phosphate+GTP+Mg^2+^+KP3702, (c) mannose-6-phosphate+GTP+Mg^2+^+KP3703, (d) mannose-6-phosphate+GTP+Mg^2+^+KP3702+KP3703. **C,** Gmd (KP3711) reaction buffers containing: (a) GDP+Mg^2+^ (b) GDP-mannose+Mg^2+^, (c) GDP-mannose+Mg^2+^+KP3711. **D,** WcaG and Gmd reaction buffers containing: (a) GDP-mannose+NADPH+KP3711+KP3709, (b) GDP-mannose and NADP^+^ standard, (c) GDP-fucose standard. **E,** WcaH (KP3708) reaction buffers containing: (a) GDP+Mg^2+^, (b) GDP-mannose+Mg^2+^, (c) GDP-mannose+Mg^2+^+KP3708. **F,** Gnd (KP3704) reaction buffers containing: (a) NADP^+^, (b) gluconate-6-phosphate+NADP^+^, (c) gluconate-6-P+NADP^+^+KP3704.
(EPS)

Figure S3 **Verification of GDP-fucose in** **Fig. 3B** **by mass spectrometry obtained on a LTQ mass spectrometer.** The peak at 588.1 is GDP-fucose, which is clearly present in Δ3715, minimally detectable in Δ3712, and absent in Δ3709.
(EPS)

Figure S4 **Growth curves of wild type and mutants in the presence of antibiotics.** **A,** tetracycline (0.5 µg/ml). **B,** sulfamethoxazole (500 µg/ml). **C,** ciprofloxacin (0.025 µg/ml). **D,** geneticin (12.5 µg/ml).
(EPS)

Table S1 **Kinetic parameters for KP3701 (UgD) and KP3704 (Gnd).**
(EPS)

Table S2 **The enzyme specificity test for ManC (KP3703).**
(EPS)

Table S3 **The enzyme specificity test for GalF (KP3726).**
(EPS)

Table S4 **Q-PCR results of non-silencing effect mutants.**
(EPS)

Table S5 **Gene annotation of NTUH K2044 from kp3689 to kp3769.**
(EPS)

Table S6 **Gene expression results of mutants strains at different temperature growth condition.**
(EPS)

Table S7 **Gene expression results of mutants strains at stationary phase (OD600: 2.0).**
(EPS)

Table S8 **Primers used for cloning the KO construct and complement plasmid.**
(EPS)

Table S9 **Primers used for quantitative PCR.**
(EPS)

## References

[1] Whitfield C (2006). Biosynthesis and assembly of capsular polysaccharides in Escherichia coli.. Annu Rev Biochem.

[2] West NP, Sansonetti P, Mounier J, Exley RM, Parsot C (2005). Optimization of virulence functions through glucosylation of Shigella LPS.. Science.

[3] Rahn A, Whitfield C (2003). Transcriptional organization and regulation of the Escherichia coli K30 group 1 capsule biosynthesis (cps) gene cluster.. Mol Microbiol.

[4] Brisse S, Fevre C, Passet V, Issenhuth-Jeanjean S, Tournebize R (2009). Virulent clones of Klebsiella pneumoniae: identification and evolutionary scenario based on genomic and phenotypic characterization.. PLoS ONE.

[5] Bina XR, Provenzano D, Nguyen N, Bina JE (2008). Vibrio cholerae RND family efflux systems are required for antimicrobial resistance, optimal virulence factor production, and colonization of the infant mouse small intestine.. Infect Immun.

[6] Podschun R, Ullmann U (1998). Klebsiella spp. as nosocomial pathogens: epidemiology, taxonomy, typing methods, and pathogenicity factors.. Clin Microbiol Rev.

[7] Woodford N, Tierno PM, Young K, Tysall L, Palepou MF (2004). Outbreak of Klebsiella pneumoniae producing a new carbapenem-hydrolyzing class A beta-lactamase, KPC-3, in a New York Medical Center.. Antimicrob Agents Chemother.

[8] Nordmann P, Cuzon G, Naas T (2009). The real threat of Klebsiella pneumoniae carbapenemase-producing bacteria.. Lancet Infect Dis.

[9] Pan YJ, Fang HC, Yang HC, Lin TL, Hsieh PF (2008). Capsular polysaccharide synthesis regions in Klebsiella pneumoniae serotype K57 and a new capsular serotype.. J Clin Microbiol.

[10] Ko WC, Paterson DL, Sagnimeni AJ, Hansen DS, Von Gottberg A (2002). Community-acquired Klebsiella pneumoniae bacteremia: global differences in clinical patterns.. Emerg Infect Dis.

[11] Yang CC, Yen CH, Ho MW, Wang JH (2004). Comparison of pyogenic liver abscess caused by non-Klebsiella pneumoniae and Klebsiella pneumoniae.. J Microbiol Immunol Infect.

[12] Lederman ER, Crum NF (2005). Pyogenic liver abscess with a focus on Klebsiella pneumoniae as a primary pathogen: an emerging disease with unique clinical characteristics.. Am J Gastroenterol.

[13] Chung DR, Lee SS, Lee HR, Kim HB, Choi HJ (2007). Emerging invasive liver abscess caused by K1 serotype Klebsiella pneumoniae in Korea.. J Infect.

[14] Fung CP, Hu BS, Chang FY, Lee SC, Kuo BI (2000). A 5-year study of the seroepidemiology of Klebsiella pneumoniae: high prevalence of capsular serotype K1 in Taiwan and implication for vaccine efficacy.. J Infect Dis.

[15] Chuang YP, Fang CT, Lai SY, Chang SC, Wang JT (2006). Genetic determinants of capsular serotype K1 of Klebsiella pneumoniae causing primary pyogenic liver abscess.. J Infect Dis.

[16] Barker SA, Brimacombe JS, Eriksen JL, Stacey M (1963). Capsular polysaccharide of Klebsiella pneumoniae type A (strain 1265).. Nature.

[17] Zamze S, Martinez-Pomares L, Jones H, Taylor PR, Stillion RJ (2002). Recognition of bacterial capsular polysaccharides and lipopolysaccharides by the macrophage mannose receptor.. J Biol Chem.

[18] Chou HC, Lee CZ, Ma LC, Fang CT, Chang SC (2004). Isolation of a chromosomal region of Klebsiella pneumoniae associated with allantoin metabolism and liver infection.. Infect Immun.

[19] Wu K-M, Li L-H, Yan J-J, Tsao N, Liao T-L (2009). Genome Sequencing and Comparative Analysis of Klebsiella pneumoniae NTUH-K2044, a Strain Causing Liver Abscess and Meningitis.. J Bacteriol.

[20] Link AJ, Phillips D, Church GM (1997). Methods for generating precise deletions and insertions in the genome of wild-type Escherichia coli: application to open reading frame characterization.. J Bacteriol.

[21] Franklin NC, Luria SE (1961). Transduction by bacteriophage P-1 and the properties of the lac genetic region in E. coli and S. dysenteriae.. Virology.

[22] Majdalani N, Gottesman S (2005). The Rcs phosphorelay: a complex signal transduction system.. Annu Rev Microbiol.

[23] Dambach MD, Winkler WC (2009). Expanding roles for metabolite-sensing regulatory RNAs.. Curr Opin Microbiol.

[24] Narberhaus F, Waldminghaus T, Chowdhury S (2006). RNA thermometers.. FEMS Microbiol Rev.

[25] Vogel J, Bartels V, Tang TH, Churakov G, Slagter-Jager JG (2003). RNomics in Escherichia coli detects new sRNA species and indicates parallel transcriptional output in bacteria.. Nucleic Acids Res.

[26] Nikaido H (1996). Multidrug efflux pumps of gram-negative bacteria.. J Bacteriol.

[27] Wu JH, Wu AM, Tsai CG, Chang XY, Tsai SF (2008). Contribution of fucose-containing capsules in Klebsiella pneumoniae to bacterial virulence in mice.. Exp Biol Med (Maywood).

[28] Lewis AL, Nizet V, Varki A (2004). Discovery and characterization of sialic acid O-acetylation in group B Streptococcus.. Proc Natl Acad Sci U S A.

[29] Lewis AL, Cao H, Patel SK, Diaz S, Ryan W (2007). NeuA sialic acid O-acetylesterase activity modulates O-acetylation of capsular polysaccharide in group B Streptococcus.. J Biol Chem.

[30] Bergfeld AK, Claus H, Lorenzen NK, Spielmann F, Vogel U (2009). The polysialic acid-specific O-acetyltransferase OatC from Neisseria meningitidis serogroup C evolved apart from other bacterial sialate O-acetyltransferases.. J Biol Chem.

[31] Liu TW, Ho CW, Huang HH, Chang SM, Popat SD (2009). Role for {alpha}-L-fucosidase in the control of Helicobacter pylori-infected gastric cancer cells.. Proc Natl Acad Sci U S A.

[32] Coyne MJ, Reinap B, Lee MM, Comstock LE (2005). Human symbionts use a host-like pathway for surface fucosylation.. Science.

[33] Yamamoto M, Sato S, Hemmi H, Sanjo H, Uematsu S (2002). Essential role for TIRAP in activation of the signalling cascade shared by TLR2 and TLR4.. Nature.

[34] Mizuta K, Ohta M, Mori M, Hasegawa T, Nakashima I (1983). Virulence for mice of Klebsiella strains belonging to the O1 group: relationship to their capsular (K) types.. Infect Immun.

[35] Reed LJ, Muench H (1938). A simple method of estimating fifty percent endpoints. .. Am J Hyg.

[36] Hsieh PF, Lin TL, Lee CZ, Tsai SF, Wang JT (2008). Serum-induced iron-acquisition systems and TonB contribute to virulence in Klebsiella pneumoniae causing primary pyogenic liver abscess.. J Infect Dis.

[37] Morona JK, Morona R, Paton JC (1997). Characterization of the locus encoding the Streptococcus pneumoniae type 19F capsular polysaccharide biosynthetic pathway.. Mol Microbiol.

[38] Chang KC, Ho SW, Yang JC, Wang JT (1997). Isolation of a genetic locus associated with metronidazole resistance in Helicobacter pylori.. Biochem Biophys Res Commun.

