# Supplementary material for: Functions of Some Capsular Polysaccharide Biosynthetic Genes in Klebsiella pneumoniae NTUH K-2044
Source: PLoS One. 2011 Jul 12;6(7):e21664. doi: 10.1371/journal.pone.0021664 (PMC3134468; doi:10.1371/journal.pone.0021664)
Supplement: Results S1 — Characterization of enzymes for the synthesis of UDP-glucose (UDP-Glc), UDP-glucuronic acid (UDP-GlcA), UDP-galacturonic acid (UDP-GalA), GDP-mannose (GDP-Man) and GDP-fucose (GDP-Fuc). (DOC) [file pone.0021664.s001.doc]

**Supporting Information**

**Functions of Some Capsular Polysaccharide Biosynthetic Genes in *Klebsiella pneumoniae* NTUH K-2044**

**Jin-Yuan Ho,** **Tzu-Lung Lin, An-Ning Cheng, Chun-Yen Li, Arwen Lee, Ming-Chuan Chen, Shih-Hsiung Wu, Jin-Town Wang, Tsung-Lin Li, and Ming-Daw Tsai**

**Supporting Results**

**Characterization of enzymes for the synthesis of UDP-glucose (UDP-Glc), UDP-glucuronic acid (UDP-GlcA), UDP-galacturonic acid (UDP-GalA), GDP-mannose (GDP-Man) and GDP-fucose (GDP-Fuc)**. Ugd, a putative hexose dehydrogenase, was assayed against UDP-Glc in the presence of NADP^+^ (**Figure S1A line d**). The conversion of UDP-GlcA from UDP-Glc with the concomitant production of NADPH from NADP^+^ concluded that Ugd is a hexose dehydrogenase. In addition, Uge was considered responsible for the formation of UDP-GalA, another dinucleotide sugar, from UDP-GlcA. The activity was confirmed as UDP-GalA was formed only in the presence of Uge (**Figure S1B line b**). However, UDP-GalA is not a component sugar in CPS. It may instead be a component in other cellular built-up such as O-antigen. Uge therefore should not be counted as a member in the *cps* gene cluster in spite of being alongside Ugd.

ManB, ManC, WcaH, Gnd, WcaG, and Gmd were considered as a cohort of enzymes for the regulation and synthesis of GDP-Man and GDP-Fuc. Reaction solutions containing mannose-1-P and GTP were carried out to test the GDP-Man phosphorylase activity of ManC **(Figure S2A line d)**. GDP-Man was identified from the LC trace in which the reaction product and the GDP-Man standard had the same retention time and the mass profiles. ManC was confirmed to be GDP-Man phosphorylase. ManB was first assayed for its phosphomannomutase activity by a coupled reaction with ManC in the presence of mannose-6-P and UTP (**Figure S2B line d**). The formation of GDP-Man was observed in the ManB and ManC coupled reaction but not in the ManC alone reaction. This concluded ManB as a phosphomannomutase. Gmd, a 4-keto-6-deoxy mannose dehydratase homologue, was tested for its capability converting GDP-Man to GDP-4-keto-6-deoxy mannose, an intermediate toward GDP-Fuc. Reactions were carried out in solutions containing GDP-Man with or without NAD^+^; a new peak emerged at 20 min that showed the same mass unit as GDP-4-keto-6-deoxy mannose **(Figure S2C line c)**. Furthermore, when WcaG, a putative GDP-Fuc synthase, was added into, the peak disappeared and a new peak appeared **(Figure S2D line a)**. This new product was identified to be GDP-Fuc. As a result, Gmd and WcaG were determined to be GDP-Man 4,6-dehydrotase and GDP-Fuc synthase, respectively. WcaH, a putative GDP-mannose mannosyl hydrolase, was characterized, when GDP-Man in solution decreased and GDP increased in the presence of WcaH **(Figure S2E line c)**. WcaH was considered involved in the regulation of the levels of GDP-Man and GDP-Fuc in response to the physiological alteration. Gnd, a putative gluconate-6-phosphate dehydrogenase, was also characterized. Gnd was assayed in the presence of gluconate-6-phosphate and NADP^+^ **(Figure S2F line c)**. The net formation of NADPH supports that Gnd is gluconate-6-phosphate dehydrogenase. In our Q-PCR results, we found that the expression of a Wzf, another dehydrogenase outside of the *cps* gene cluster, was increased in the Δgnd strain. Thus, both Gnd and Wzf, two major enzymes for NADPH productions, could be functioning in the CPS synthesis.
